# Flesh Diet for Children

**Published:** 1887-10

**Authors:** 


### FLESH DIET FOR CHILDREN.

Without entering into the general question of vegetarianism, or discussing the propriety of the total rejection of meat, as an article of diet, we wish to call attention to the fact, that a flesh diet is eminently improper for young children. The evidences of this fact are very numerous and indisputable. We shall not, at this time, enter into a full discussion of the evidences, but we wish to call attention to the following from the pen of Dr. D. M. Camman, of New York, who is entitled to speak authoratively on the subject of children's diet :—

"Are young children under seven or eight years of age best reared on a diet, composed largely of meat? or is the best result attained by giving largely of milk, vegetables, and cereals, and reserving meat for a later period? It may be reasonably supposed that the delicate mucous membrane of the child, and the temporary teeth, which are called milk teeth, are best suited to the latter form of diet. It has been found, too, that the gastro-intestinal complaints so prevalent with children, especially in the summer, are infrequent in those cases where meat has been ommitted from the dietary, and milk constitutes the principal food. Dr. Clonston, after saying that he agrees with Dr. Keith, who has preached an anti-flesh crusade in the bringing up of all children to eight or ten years of age, continues, 'I believe that by a proper diet and regimen, more than in any other way, we can fight against and counteract inherited neurotic tendencies in children, and tide them safely over the periods of puberty and adolescence."

A few facts are worth more than any *a priori* reasoning. These may be gleaned from the experience gained in the use of the following dietary of the Orphans' Home and Asylum of New York, which has been used without any material change for the past twenty-seven years. During this time the rarity of disturbances of the digestive organs has been remarkable, and the recovery of the children suffering from other diseases, such as scarlet fever, has been exceptionally rapid. Especially would it seem that this diet was suitable for those who show any tendency to disturbances of the nervous system, either inherited or acquired. The death-rate in the institution has been extremely low. The number of children is about one hundred and forty-five. Children are not admitted under three years of age."

In the above-named institution, children over eight years of age are allowed an occasional taste of meat at dinner, but are not allowed milk at the same meal. There is no physiological change in the child at the age of eight years, which renders the use of flesh food either necessary or more wholesome than at an earlier age, although it is undoubtedly true that a moderate allowance of flesh may be taken at this period of life, with less absolute danger than at an earlier period. It is the candid opinion of the writer, after many years' personal experience and observation, that at least the great majority of large children, as well as small ones, do a great deal better upon a non-flesh diet.—*Good Health.*

